# Intracranial Hemorrhage in Hospitalized Patients Following Percutaneous Coronary Intervention: A Large Cohort Analysis from a Single Center

**DOI:** 10.3390/diagnostics13142422

**Published:** 2023-07-20

**Authors:** Cheng Yang, Yong-Gang Sui, Bin-Cheng Wang, Yan-Lu Xu, Na-Qiong Wu, Yong-Jian Wu, Jian-Jun Li, Jie Qian

**Affiliations:** Department of Cardiology, Fu Wai Hospital, National Center for Cardiovascular Diseases, Chinese Academy of Medical Sciences and Peking Union Medical College, Beijing 100037, China; yangcheng_fw@163.com (C.Y.); fwsyg999@163.com (Y.-G.S.); xiaoliang-24@163.com (B.-C.W.); billyyanlu@aliyun.com (Y.-L.X.); fuwainaqiongwu@163.com (N.-Q.W.); yongjianwu_nccd@163.com (Y.-J.W.)

**Keywords:** symptomatic intracranial hemorrhage, percutaneous coronary intervention, coronary artery disease, incidence, clinical characteristics

## Abstract

Background: There are several reports on the prevalence and characteristics of intracranial hemorrhage (ICH) following percutaneous coronary intervention (PCI), which is a rare but severe complication with high mortality. However, the clinical landscapes of computed tomography (CT)-confirmed, symptomatic ICH in hospitalized patients are not fully characterized. Methods: Among 121,066 patients receiving PCI treatment in the Fu Wai Hospital between 2013 and 2022, there were 18 CT-defined, symptomatic patients with ICH occurring during post-PCI hospitalization. Symptomatic ICH was defined as clinical suspicion of hemorrhage and/or new focal neurological signs. We analyzed ICH timing, clinical and imaging features, and subsequent outcomes. Results: Overall, in this retrospective analysis, the incidence of CT-defined, symptomatic ICH was 0.015% (18/121,066). More than half of the cases (55.6%) occurred within the first 12 h following PCI. The most common initial manifestation of ICH patients was disturbance of consciousness. Thirteen patients (72.2%) had a hematoma volume ≥ 30 cm^3^. Additionally, the ICH was observed in the cerebral lobe (66.7%), cerebellum (22.2%), and the basal ganglia and thalamus (11.1%). The 90-day mortality of ICH patients undergoing PCI was very high (72.2%). Consciousness disturbance (*p* = 0.036), intracerebral hemorrhage volume > 30 mm^3^ (*p* = 0.001), and intracerebral hemorrhage originating from the infratentorial origin (*p* = 0.044) were significantly higher in patients who died. Conclusions: Symptomatic ICH events occur with a rate of around 0.015%, with significantly higher short-term mortality risk in our cohort receiving PCI, which has not yet been demonstrated in other cohorts.

## 1. Introduction

Antithrombotic therapy is the cornerstone of treatment for patients with ischemia coronary artery disease undergoing percutaneous coronary intervention (PCI), which can effectively reduce the incidence of thromboembolic events while increasing the risk of bleeding [1]. There is now solid evidence available indicating that there is a significant association between severe bleeding events and unfavorable outcomes, necessitating particular attention [2].

Intracranial hemorrhage (ICH) is the most devasting bleeding complication after PCI and has been considered a crucial safety end point in antithrombotic treatment trials. It has also been documented to be rare; as a result, it is frequently neglected, even in typical patients exhibiting the risk of bleeding. A comprehensive comprehension of PCI-related ICH will assist physicians to determine a reasonable antithrombotic strategy that balances the bleeding and ischemic risks. However, there are limited studies exploring ICH following PCI. At the individual level, the risk for ICH in patients was relatively low. In a pooled patient-level data analysis of 37 815 patients with non-ST-segment elevation acute coronary syndrome during a median follow-up of 332 days from four contemporary antithrombotic therapy trials, ICH occurred in 135 patients (0.4%) [3].On the contrary, at a population level, the total health economic and care burden of ICH associated with PCI is considerable. In Italy, the incidence rate of PCI for coronary artery diseases ranges from 120 to 178 cases per 100 000 residents per year. Furthermore, in the United States, approximately 1 to 1.2 million people are hospitalized with acute coronary diseases each year. In 2020, the global prevalence of stroke was 89.13 million cases, of which 18.88 million cases also showed a global prevalence of ICH [4,5]. As a consequence, the overall count of ICH cases following PCI may be quite substantial.

ICH following PCI is infrequent but is associated with a high mortality rate and potentially causes life-changing disabilities in those who survive [6,7,8]. Several clinical studies have demonstrated that ICH patients have a fatality rate ranging from 40% to 50% within 30 days and no or minimal trend improvements over more recent time epochs [9,10,11]. The mortality rate for ICH patients associated with antiplatelet drugs is even higher, reaching up to 60% during contemporary times [12]. ICH patients have poor prognoses and few effective treatments, but numerous models exist to predict mortality and functional outcomes after ICH. The ICH score is the most commonly used prognostic scale, combining demographic and clinical information. It reports that several components, including age >80 years, ICH volume greater than 30 mm^3^, infratentorial origin of hematoma, presence of intraventricular hemorrhage and Glasgow Coma Scale score, were associated with 30-day mortality after ICH [13]. Additionally, the use of antithrombotic drugs also contributed to unfavorable outcomes among patients with ICH.

There is still a lack of effective therapies to improve the prognosis of ICH patients. Hence, prevention is undoubtedly the best approach. Therefore, physicians and patients need to acquire more accurate information regarding clinical characteristics of ICH after PCI during hospitalization and its severity and outcomes, consequently, assisting to establish doctor–patient shared decision making. Previous large-scale studies (e.g., from the Bleeding complications in a Multicenter registry of Acute Coronary Syndrome (BleeMACS) project) have studied the incidence, predictors, and prognostic impact of intracranial bleeding within the first year after an acute coronary syndrome in patients treated with PCI [14].

Nevertheless, there is a paucity of information existing on the clinical characteristics of in-hospital ICH after PCI, the timing of ICH related to the procedure, and the subsequent clinical outcomes. The objectives of this real-world, retrospective study, hence, were to describe the incidence, clinical, and imaging characteristics and mortality of peri-procedural ICH patients from a large single center cohort.

## 2. Materials and Methods

### 2.1. Study Design and Population

A total of 121,066 patients aged ≥18 years who presented with stable angina, unstable angina, or acute myocardial infarction receiving PCI treatment between January 2013 and December 2022 at the Fu Wai Hospital were retrospectively screened. All information for patients who underwent PCI for coronary artery disease was prospectively recorded in a dedicated database in Fu Wai Hospital. The recorded information included baseline demographics, diagnoses, cardiovascular risk factors, angiographic and PCI procedural details, medication usage (prior-, during-, or post-procedure), hospitalization course, as well as laboratory examinations. Inclusive criteria were cranial computed tomography (CT)-confirmed symptomatic ICH patients who had undergone PCI for the treatment of coronary artery disease. For patients with traumatic intracranial hemorrhage, patients with incomplete major data, or those who only had coronary angiography (without PCI treatment) were excluded from this study. For patients receiving PCI treatment more than one time within the same year of the index day, only one procedure was counted.

### 2.2. Definitions of Diseases

ICH refers to any bleeding within the intracranial vault, including the brain parenchyma and surrounding meningeal spaces. Symptomatic ICH was defined as clinical suspicion of hemorrhage and/or new focal neurologic signs. Cranial CT scans were used to confirm the diagnosis of ICH. Routine brain CT screening was not performed after PCI. Additionally, the onset time was defined as the first occurrence of abnormal symptoms that were observed by the medical team after PCI and while hospitalized in the cardiology department. ICH location and volume were adjudicated by both radiologists and neurologists. Subarachnoid hemorrhage (SAH) and intraventricular hemorrhage (IVH) were not counted when calculating the ICH volume, which consisted of the volume of intracerebral hemorrhage. The ICH volume was calculated on the brain non-contrast CT scan according to the ABC/2 method, in which A represented the greatest diameter on the largest hemorrhage slice, B represented the diameter at a 90° angle to A, and C represented the approximate number of 10 mm slices on which the hematoma was measured [15]. Consequently, ICH was classified into two categories as small (<30 cm^3^) or large (≥30 cm^3^), as described in previous studies [13]. Renal insufficiency was defined as a creatinine clearance of <60 mL/min, as was estimated by the Cockcroft–Gault equation.

### 2.3. Clinical Outcomes

The study outcome was all-cause death measurements recorded at 90 days of index ICH. All-cause death was considered as cerebrovascular death and cardiovascular death unless a non-cerebrovascular death or non-cardiovascular death could be definitively identified. The start of follow-up for ICH patients was defined as the date of ICH of the case. Clinical outcome information was obtained by telephone contacts at 90 days. All eligible patients provided electronic informed consent by telephone interview follow up.

### 2.4. Statistical Analysis

Continuous variables were presented as mean ± standard deviation or median (25th–75th percentiles). Categorical variables were presented as observed frequencies and proportions (%). Furthermore, differences between groups were assessed by chi-square (or Fisher’s exact test when the expected cell value was <5) for categorical variables. Continuous data were compared by the Student t-test or the Mann–Whitney U test, as determined by the distribution of analyzed variables. A two-sided *p* value of <0.05 was considered statistically significant. Statistical analysis was performed using SPSS 25.0 (IBM, Armonk, NY, USA).

## 3. Results

### 3.1. General Characteristics

A total of 18 patients (0.015%) suffered ICH following PCI. Clinical and procedural demographics are presented in Table 1. The mean age of the patients was 64.56 ± 11.82 years and 72.2% were men. In addition, twelve patients (66.7%) had hypertension, two of whom had poorly controlled blood pressure (systolic blood pressure ≥140 mmHg and/or diastolic blood pressure ≥90 mmHg). Regarding other cardiovascular risk factors, 22.2% of the patients had diabetes mellitus and 88.9% had dyslipidemia.

Other comorbid conditions included prior ischemia stroke or TIA in 2 patients (11.1%), renal insufficiency in 2 patients (11.1%), and anemia in 3 patients (16.7%). A total of four patients (22.2%) were diagnosed with myocardial infarction. In relation to procedure information, 14 patients (77.8%) underwent elective PCI and 15 patients (83.3%) had stents implanted.

Of all the patients with an indication to receive dual antiplatelet therapy (DAPT) at baseline (prior to ICH onset), most (72.2%) received DAPT with aspirin plus Clopidogrel. Overall, 16 patients (88.9%) received unfractionated heparin during PCI and the remaining 2 patients (11.1%) received bivalirudin.

### 3.2. Imaging Characteristics

**Figure 1:**
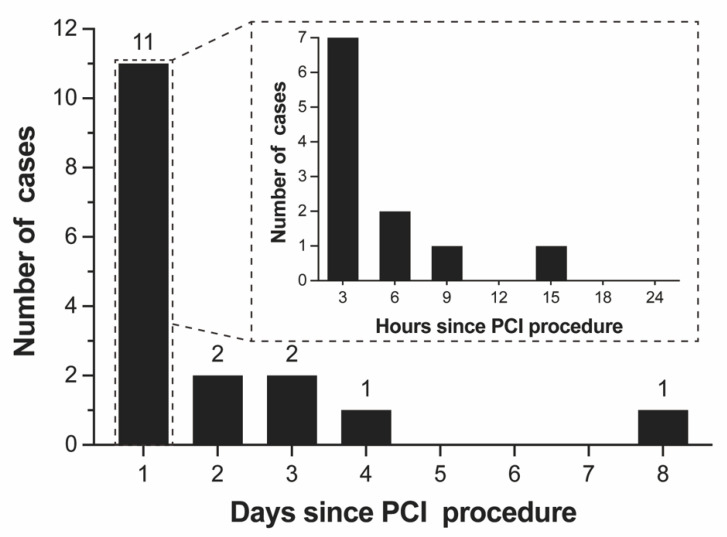
Timing of ICH in hospitalization after PCI. ICH = intracranial hemorrhage; PCI = percutaneous coronary intervention.

During hospitalization, the median time to ICH was 7 h (interquartile range, 2–52 h) after PCI. Of note, more than half (*n* = 10, 55.6%) of the cases occurred within the first 12 h after PCI and more than one-third (*n* = 7, 38.9%) occurred within 3 h of PCI, as shown in Figure 1. The clinical and imaging characteristics of 18 patients with ICH are represented in Table 2. The most common initial manifestation of ICH patients was disturbance of consciousness (*n* = 9, 50.0%), followed by headaches (*n* = 6, 33.3%), and focal neurological signs (*n* = 5, 27.8%).

**Figure 2:**
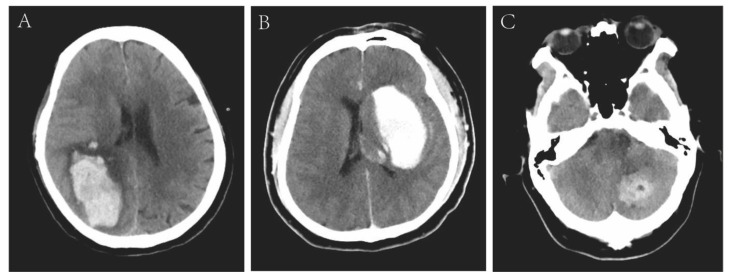
Representative computed tomography images of patients with intracranial hemorrhage. The representative patient (No. 9) had disturbance of consciousness at 72 h after percutaneous coronary intervention (PCI), with bleeding in the frontal, parietal, and temporal lobes (**A**). Patient (No. 3) had focal neurological dysfunction at 2 h after PCI, with bleeding in the basal ganglia and thalamus (**B**). Patient (No. 8) had headache and vomiting at 72 h after PCI, and bleeding site was in the left cerebellar hemisphere (**C**).

All 18 patients received brain CT scans. The median time from the onset of symptoms to the first CT scan was 44 min (interquartile range, 33–103 min). The representative CT images from three patients are shown in Figure 2. The lobe, cerebellum, and deep basal ganglia and thalamus were affected in 12 patients (66.7%), 4 patients (22.2%), and 2 patients (11.1%), respectively. Among them, four patients (22.2%) combined with SAH and two patients (11.1%) combined with IVH (Table 3). The mean ICH volume was 65.83 ± 53.34 cm^3^, and 13 patients (72.2%) had hematoma volume ≥30 cm^3^.

### 3.3. Clinical Outcomes

A total of 18 patients (100%) completed 90-day clinical follow up. The 90-days mortality rate was 72.2% for a median of 2.5 days (interquartile range, 1–80 days), with most deaths occurring within 3 days (*n* = 10, 76.9%). The 90-day mortality rate for ICH occurring within 12 h after PCI was 90%. Moreover, the 90-day mortality rate was 92.3% for patients with an ICH volume of more than 30 cm^3^. Furthermore, the 90-day mortality was 100% for patients with disturbance of consciousness as the initial symptom. According to the outcome, the proportion of patients with disturbance of consciousness (*p* = 0.036), ICH volume >30 mm^3^ (*p* = 0.001), and infratentorial origin of the ICH (*p* = 0.044) were significantly higher in the death group than in the survival group (Table 2).

## 4. Discussion

ICH is a rare but lethal complication in patients who received PCI. In this retrospective study from a large Chinese cohort, we found that (1) ICH most commonly occurred within 12 h of PCI, which acts as the first reported finding in the literature using 12 h as a procedural-related time point; (2) the cerebral lobe was the most frequent bleeding site, which is quite different from hypertensive intracerebral hemorrhage; (3) current analysis suggested that coronary artery disease patients accompanied with hypertension are prone to ICH even in the patients with well-controlled status; (4) the 90-day mortality was extremely high (72.2%). These findings may provide additional information regarding ICH after PCI.

Our study derived data from the national center for cardiovascular diseases with the largest number of PCI procedures over the past 10 years in China. The incidence of ICH in patients after PCI during hospitalization was 0.015%, which was slightly lower than in previous studies. Myint et al. analyzed 560,439 patients undergoing PCI between 2007 and 2012 in the British Cardiovascular Intervention Society (BCIS) database and found that the incidence of intracerebral hemorrhage after PCI during hospitalization was 0.02% [16]. However, few studies have been published on ICH after PCI and, if any occur, they are usually conducted during follow-up after discharge; whereas, rare studies have been conducted on ICH patients during hospitalization. The investigators of BleeMACS reported an ICH rate of 0.27% in the first year post discharge [14]. A recent study with a long-term follow-up period analyzed data from the Korean National Health Insurance Service database showed an incidence rate of ICH after PCI of 3.32 cases per 1000 person-years during a median follow-up of 5.6 years [10]. Therefore, the incidence of ICH after PCI is low, and the aforementioned studies are limited by the small number of ICH cases, making it difficult to develop pertinent clinical practice studies.

Our data indicated that a 72.2% rate of ICH was observed in patients within 12 h of PCI, with more than half of ICH occurring within 48 h. Accordingly, ICH most probably occurred instantly after the PCI, and the 12 h incidence rate of ICH overweighs the incidence rate of other in-hospital post-PCI periods. For instance, in a study of 5372 patients undergoing PCI for STEMI, Jeffrey et al. showed that all hemorrhagic strokes (eight cases) occurred within 48 h after PCI, suggesting that the risk of early ICH after PCI is higher [17]. This observation suggested that dual antiplatelet exposure and periprocedural anticoagulation therapies make at-risk patients more susceptible to ICH. In the meta-analysis with data from four antithrombotic therapy trials, a large number of antithrombotic agents were independent predictors of ICH (HR per each additional agent, 2.06; 95% CI, 1.49 to 2.84) [3]. As a matter of fact, it is critical to be aware of the life-threatening complications of ICH in the early stages during the period of PCI, particularly in patients at high risk of bleeding. Specifically, perioperative antithrombotic strategies, including antiplatelet agents and anticoagulation selection, dosage, timing, and duration, shall be properly and thoroughly evaluated.

Hypertension was the strongest attribute risk factor for spontaneous intracerebral hemorrhage in most population-based studies [18]. It could cause deep perforating vasculopathy or arteriolosclerosis; therefore, the hemorrhage site is often located in the basal ganglia and thalamus [12]. In contrast to this, our analysis demonstrated that the most common location of ICH following PCI were cerebral lobes, followed by the cerebellum. Only two patients had bleeding sites in the basal ganglia and thalamus. Notably, one-third of all patients with ICH in our study did not suffer from hypertension, and 83% (10/12) of those with hypertension had well-controlled blood pressure, which suggested that patients with coronary artery disease accompanied by hypertension were vulnerable to ICH even in the long-term well-controlled blood pressure group. The reason for this observation might relate to periprocedural antiplatelet and anticoagulant therapy. Nevertheless, our current study did not reveal a significant correlation between the hematoma location and clinical outcomes. In the contemporary era of PCI, further study is needed to explore the impact of bleeding sites on the outcome of ICH related to catheter intervention.

The mortality rate for ICH patients was as high as 72.2%. In previous studies, the mortality rate for spontaneous ICH has been estimated to be between 30% and 40% and has remained stable over the past 20 years [19,20,21]. In the study analyzing ICH patients (107 cases) after PCI in England and Wales, the 30-day mortality was 48% [22]. Our study showed that approximately three-fourths of the patients died within 90 days after the ICH events, which is higher than in prior studies. It is also worth pointing out that patients with ICH onset within 12 h after PCI had a 90-day mortality rate of 90%. In addition, an exploratory analysis of the TICH-2 (Tranexamic Acid in Intracerebral Hemorrhage-2) trial demonstrated that pre-ICH antiplatelet therapy was associated with hematoma expansion and unfavorable outcomes at 90 days [23]. Hematoma expansion occurs early after ICH and is an independent determinant of mortality [24]. In another study, among those who underwent the initial CT scan within 3 h of ICH onset, hematoma expansion after 24 h of ICH onset was extremely rare [25], which might be the reason for the high mortality rate in the early stage of ICH onset. However, in our study, due to the rapid progression of the disease and the quick reduction in the level of consciousness, it was not feasible to perform follow-up CT scans. Given the poor prognosis, high mortality, and expensive healthcare costs of patients with ICH, it is crucial to provide more outcome information. This benefits in determining treatment strategies and clinical decision making. Our work provides insights into the outcomes associated with this rare but devastating complication of PCI to neurological physicians who may not frequently encounter such patients, i.e., patients frequently treated with potent antiplatelet and anticoagulant therapies, which are necessitated during the PCI procedure.

Our study has several limitations. Firstly, these analyses were derived from a retrospective study and, therefore, are subject to the inherent limitations of observational databases. Secondly, out-of-hospital and asymptomatic ICH were not included in our study. Thirdly, a small sample size and single center study is another limitation. Finally, our work did not explore the etiology of ICH patients, such as arteriovenous malformations, aneurysms, cerebral amyloid angiopathy (CAA), etc. Because of the high fatality rate and rapid progression of this illness, only initial brain CT scanning was carried out and no further imaging examinations, including digital subtraction (DSA) and magnetic resonance imaging (MRA), were performed since it is difficult to pinpoint the cause of ICH. Finally, we did not have accurate figures on the total number of patients who underwent cranial CT scans after PCI.

## 5. Conclusions

Our study may address an unmet need by providing real-world evidence for the incidence, clinical characteristics, and mortality of ICH in patients undergoing PCI during hospitalization. Peri-procedural ICH events rarely occurred in patients with coronary artery disease receiving PCI. However, post-PCI patients accompanied by peri-procedural ICH events were associated with significantly higher short-term mortality risk.

## Figures and Tables

**Table 1:** Baseline and procedural characteristics of patients with ICH.

Variables	Overall (*n* = 18)	Death Group (*n* = 13)	Survival Group (*n* = 5)	*p* Value
Demographics				
Age, years	64.56 ± 11.82	64.15 ± 11.68	65.60 ± 12.60	0.976
Male	13 (72.2)	9 ((69.2)	4 (80.0)	1
BMI, kg/m^2^	25.83 ± 4.39	25.95 ± 5.09	25.54 ± 2.04	1
Medical history				
Current smoking	6 (33.3)	4 (30.8)	2 (40.0)	1
Hypertension	12 (66.7)	9 (69.2)	3 (60.0)	1
Diabetes mellitus	4 (22.2)	3 (23.1)	1 (20.0)	1
Dyslipidemia	16 (88.9)	11 (84.6)	5 (100)	1
Anemia	3 (16.7)	3 (23.1)	0 (0)	0.522
Renal insufficiency	2 (11.1)	1 (7.7)	1 (20.0)	0.490
Peripheral vascular disease	3 (16.7)	3 (23.1)	0 (0)	0.522
Prior ischemia stroke/TIA	2 (11.1)	2 (15.4)	0 (0)	1
Prior MI	2 (11.1)	2 (15.4)	0 (0)	1
Prior PCI	5 (27.8)	4 (30.8)	1 (20.0)	1
Diagnosis				0.636
Stable angina	5 (27.8)	4 (30.8)	1 (20.0)	
Unstable angina	9 (50.0)	7 (53.8)	2 (40.0)	
MI	4 (22.2)	2 (15.4)	2 (40.0)	
Procedural characteristics				
Duration of procedure, mins	81.33 ± 40.40			
Selective	14 (77.8)	11 (84.6)	3 (60.0)	0.533
Emergency	4 (22.2)	2 (15.4)	2 (40.0)	0.533
Stent implantation	15 (83.3)	12 (92.3)	3 (60.0)	0.172
Antithrombotic therapy				
Pre-procedure				1
Aspirin plus clopidogrel	13 (72.2)	9 (69.2)	4 (80.0)	
Aspirin plus ticagrelor	5 (27.8)	4 (30.8)	1 (20.0)	
During procedure				1
Unfractionated heparin	16 (88.9)	11 (84.6)	5 (100)	
Bivalirudin	2 (11.1)	2 (15.4)	0 (0)	
Post-procedure				0.132
Tirofiban	1 (5.6)	0 (0)	1 (20.0)	
LMWH	10 (55.6)	6 (46.2)	4 (80.0)	
Fondaparinux	2 (11.1)	2 (15.4)	0 (0)	
Duration of hospitalization, days	4.74 ± 2.71	5.08 ± 3.28	4.80 ± 0.84	0.782

Data are shown as mean ± standard deviation or as *n* (percentage). ICH = intracranial hemorrhage; BMI = body mass index; TIA = transient ischemia attack; MI = myocardial infarction; PCI = percutaneous coronary intervention; LMWH = low-molecular-weight heparin.

**Table 2:** ICH-related characteristics (*n* = 18).

Variables	Overall (*n* = 18)	Death Group (*n* = 13)	Survival Group (*n* = 5)	*p* Value
Clinical characteristic				
Initial symptoms				
Focal neurological signs	5 (27.8)	4 (30.8)	1 (20.0)	1
Disturbance of consciousness	8 (44.4)	8 (61.5)	0 (0)	0.036
Time to symptoms after procedure				
Median time, hours	7 (2–54)	3 (1.5–36)	48 (75–84)	0.099
Within 12 h	10 (55.6)	9 (69.2)	1 (20.0)	0.118
More than 12 h	8 (44.4)	4 (30.8)	4 (80.0)	0.118
Neuroradiologic data				
Location 1				0.109
Lobar	12 (66.7)	10 (76.9)	2 (40.0)	
Cerebellar	4 (22.2)	1 (7.7)	3 (60.0)	
Deep (basal ganglia and thalamus)	2 (11.1)	2 (15.4)	0 (0)	
Location 2				0.044
Infratentorial	4 (22.2)	1 (7.7)	3 (60.0)	
Supratentorial	14 (77.8)	12 (92.3)	2 (40.0)	
Combined with SAH	4 (22.2)	4 (30.8)	0 (0)	0.278
ICH volume, cm^3^	65.83 ± 53.34	84.17 ± 50.74	18.14 ± 20.85	0.001
Small (<30 cm^3^)	5 (27.8)	1 (7.7)	4 (80.0)	0.008
Large (≥30 cm^3^)	13 (72.2)	12 (92.3)	1 (20.0)	0.008
Brain herniation	4 (22.2)	4 (30.8)	0 (0)	0.278
Intraventricular hemorrhage	2 (11.1)	2 (15.4)	0 (0)	1
Midline shift ≥ 10 mm	6 (33.3)	6 (46.2)	0 (0)	0.114
Treatment				
Conservative medicine	17 (94.4)	13 (100)	4 (0)	0.278
Minimally invasive surgery	1 (5.6)	0 (0)	1 (20.0)	0.278

Data are shown as mean ± standard deviation, *n* (percentage), or median (IQR). SAH = subarachnoid hemorrhage; other abbreviations as defined in Table 1.

**Table 3:** Detailed information of the 18 post-PCI patients who suffered ICH.

No.	Age	Gender	Dual Antiplatelet Therapy, Aspirin Plus	During Procedural Anti-Coagulants	Time since PCI, Hours	Onset Symptoms	CT Manifestations		90-Day Clinical Outcomes
							Bleeding Site	Volume, cm^3^	
1	71	F	clopidogrel	UFH	192	DC, FNS	FL	61	Died
2	75	F	ticagrelor	UFH	3	HA, V	FL, PL	42	Died
3	52	M	clopidogrel	UFH	2	FNS	BG, TH, EC, IVH	58	Died
4	60	M	ticagrelor	UFH	1	V, FNS	TL, BG,	38	Died
5	64	M	clopidogrel	UFH	1	HA, DC	PL, OL, SS	120	Died
6	44	M	ticagrelor	UFH	48	FNS, V	FL	113	Died
7	82	F	clopidogrel	bivalirudin	2	HA, DC	TH, BG, CH	91	Died
8	68	M	clopidogrel	UFH	72	HA, V	CE	10	Survived
9	73	M	ticagrelor	UFH	72	HA, V	FL, PL, OL	54	Survived
10	67	F	clopidogrel	UFH	3	HA, V	CE	11	Survived
11	65	M	clopidogrel	UFH	96	FNS	FL, PL	2	Survived
12	45	M	clopidogrel	UFH	48	HA	CE	12	Died
13	65	F	clopidogrel	UFH	24	DC	FL, SS	70	Died
14	44	M	ticagrelor	UFH	2	DC	FL, TL, SS, CH	92	Died
15	78	M	clopidogrel	UFH	12	V	CE, CH	9	Survived
16	67	M	ticagrelor	UFH	8	DC	PL, TL, OL	94	Died
17	77	M	clopidogrel	bivalirudin	6	DC	FL, SS, EC, IVH	217	Died
18	63	M	clopidogrel	UFH	6	DC	TL, CH	82	Died

F = female; M = male; UFH = unfractionated heparin; HA = headache; V = vomiting; DC = disturbance of consciousness; FNS = focal neurological signs; FL = frontal lobe; PL = parietal lobe; TL = temporal lobe; OL = occipital lobe; BG = basal ganglia; TH = thalamus; CE = cerebellum; SS = subarachnoid space; EC = encephalocele; CH = cerebral hernia; other abbreviations as defined in Table 1.

## Data Availability

The data shown in this study are available on request from the corresponding author.
